# Supplementary material for: Spatially confined protein assembly in hierarchical mesoporous metal-organic framework
Source: Nat Commun. 2023 Feb 21;14:973. doi: 10.1038/s41467-023-36533-w (PMC9944321; doi:10.1038/s41467-023-36533-w)
Supplement: Supplementary file 1 — Supplementary Information [file 41467_2023_36533_MOESM1_ESM.pdf]

Supplementary Information

Spatially Confined Protein Assembly in Hierarchical Mesoporous Metal-Organic Framework

Xiaoliang Wang,[1,2] Lilin He,[3*] Jacob Sumner,[3] Shuo Qian,[3,4] Qiu Zhang,[3] Hugh O'Neill,[3] Yimin Mao,[5,7] Chengxia Chen,[1] Abdullah M. Al-Enizi,[6] Ayman Nafady,[6] Shengqian Ma[1*]

1. Department of Chemistry, University of North Texas, Denton, Texas 76201, United States
2. Department of Chemistry, University of South Florida, Tampa, Florida 33620, United States
3. Neutron Scattering Division, Oak Ridge National Laboratory, Oak Ridge, Tennessee 37831, United States
4. The Second Target Project of SNS, Oak Ridge National Laboratory, Oak Ridge, Tennessee 37831, United States
5. NIST Center for Neutron Research, National Institute of Standards and Technology, Gaithersburg, Maryland 20899, United States
6. Department of Chemistry, College of Science, King Saud University, Riyadh 11451, Saudi Arabia
7. Department of Materials Science and Engineering, University of Maryland, College Par, MD 202742, United States

Corresponding Authors: hel3@ornl.gov, Shengqian.Ma@unt.edu

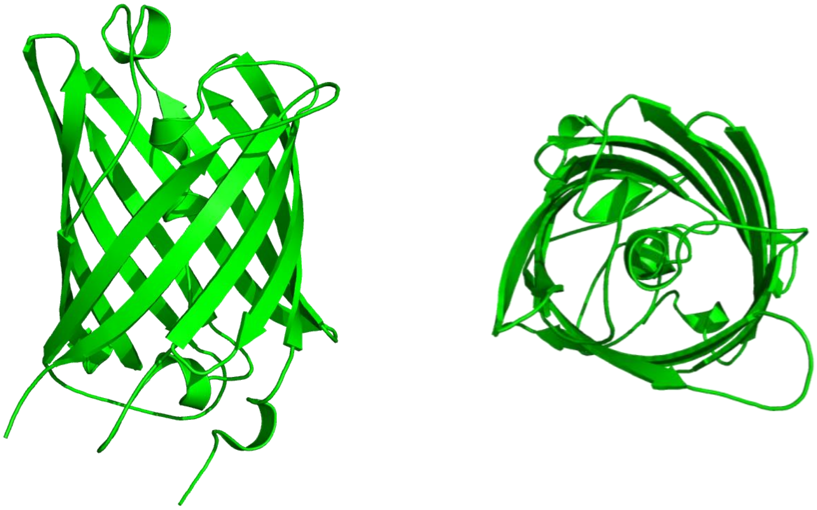

Supplementary Figure 1. The structure of GFP with a length of 4.2 nm and a diameter of about 2.4 nm (PDB entry: 1GFL).

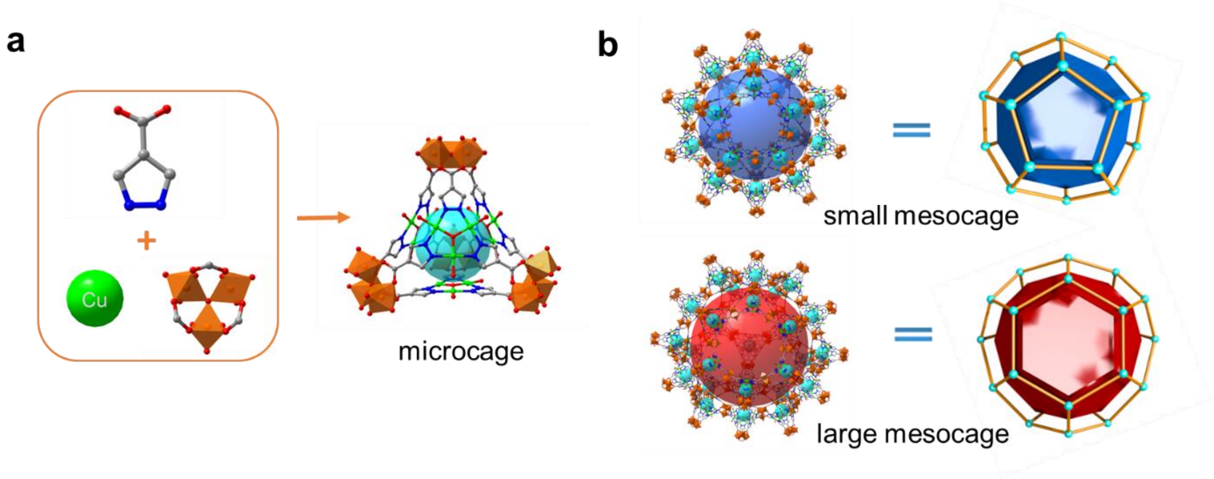

Supplementary Figure 2. Structures of MOF-919 (a) construction of microcage, (b). Structure and tiling of mesoporous cages, including small mesocage and large mesocage. The atom color code: grey for C; blue for N; brown for Al; red for O; green for Cu.

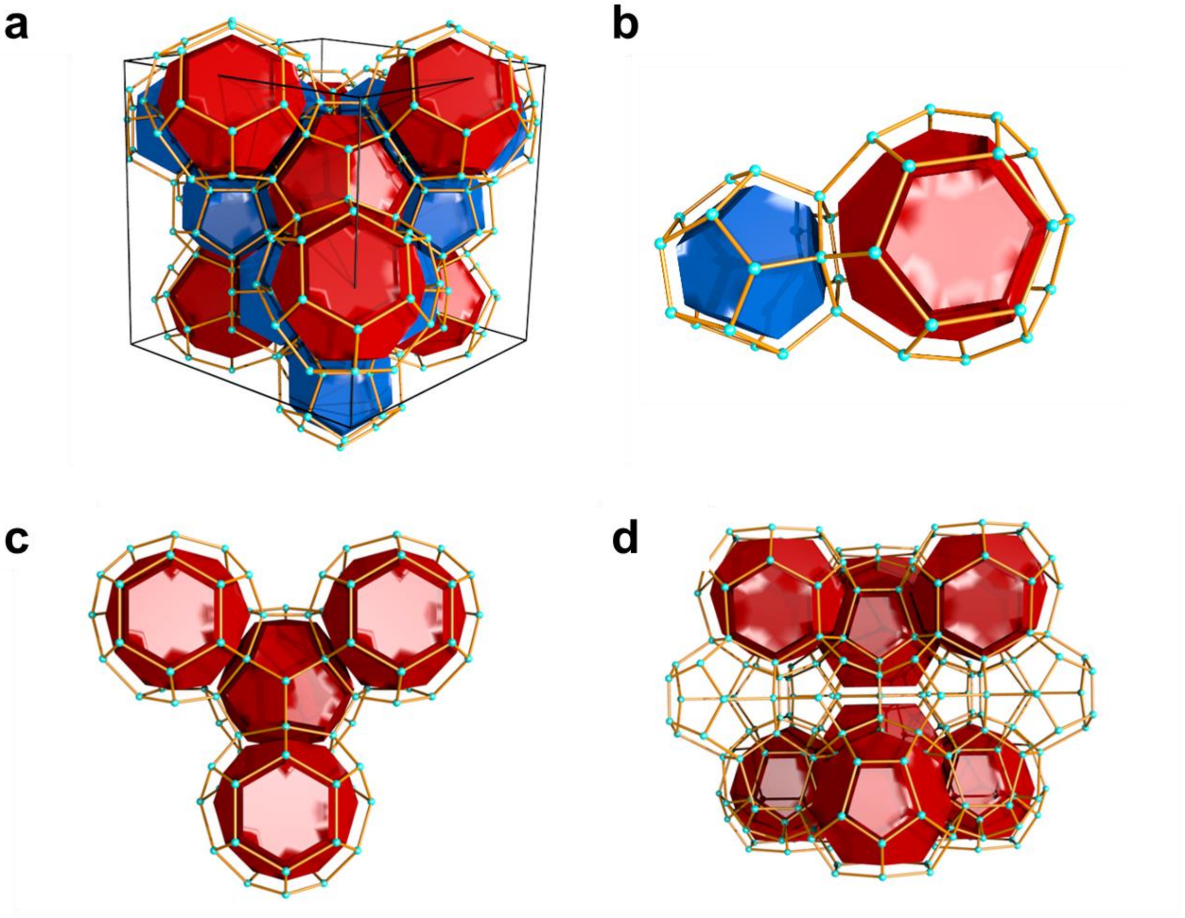

Supplementary Figure 3. (a) **MTN** zeotype topology and (b) tiling of connected small and large mesocages, and (c, d) the highlighted arrangement of large *liu* cage in the structure of MOF-919.

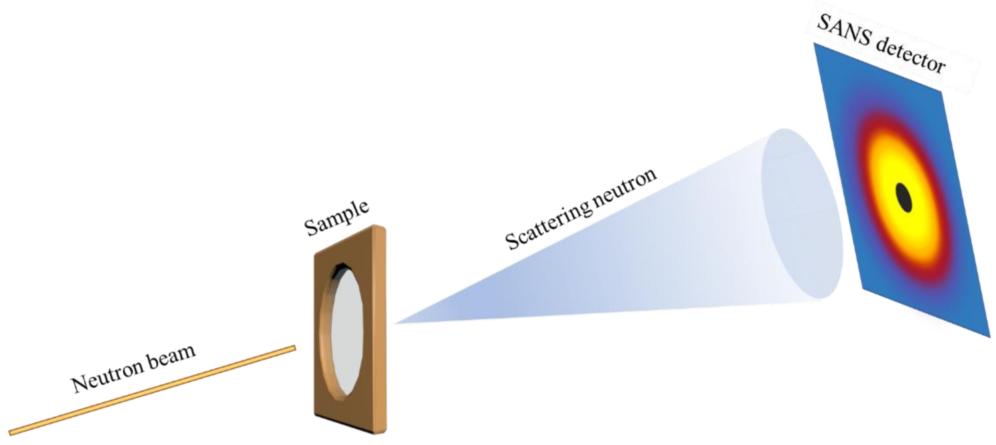

Supplementary Figure 4. Schematic of measurement for GFP loaded MOF-919 composites by SANS.

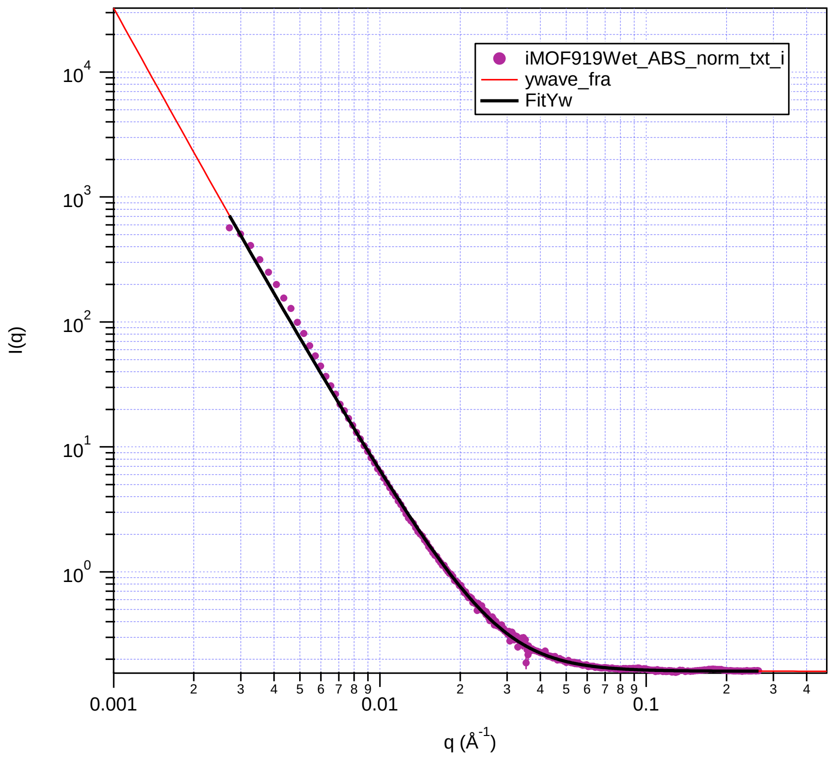

Supplementary Figure 5. Fitting of the wet MOF-919 at 50%/50% of D_2O/H_2O using the Fractal form factor.

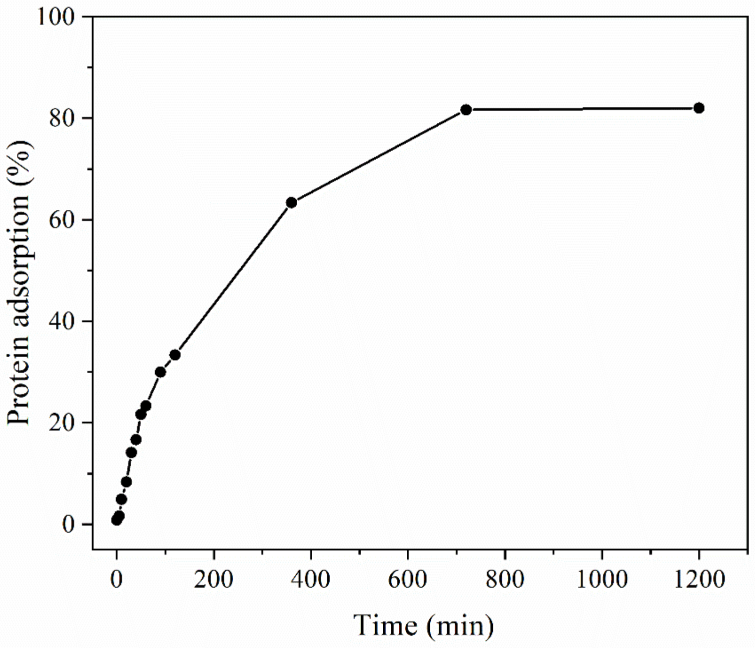

Supplementary Figure 6. The adsorption profile of h-GFP into MOF-919 in 20 mM Tris-buffer at pH 7.5.

Supplementary Table 1. The fitting parameters of unloaded MOF-919 at dry state.

	**MOF-919-Dry**
**Porod Scale**	5.63e-06
**Porod Exponent**	3.42
**Correlation Length Scale**	2445
**Correlation Length [Å]**	264.98
**Exponent m**	5.85
**Scale factor of 1st Peak**	2.81e-03
**Position of 1st Peak**	0.0902
**Std Dev of 1st Peak**	0.00889
**Scale factor of 2nd Peak**	3.49e-02
**Position of 2nd Peak**	0.0513
**Std Dev of 2nd Peak**	0.0190
**Scale factor of 3rd Peak**	8.47e-02
**Position of 3rd Peak**	0.176
**Std Dev of 3rd Peak**	0.0178
**Bkg [1/cm]**	0.1858

Supplementary Table 2. The fitting parameters of unloaded MOF-919 in the contrast matching point of 50% D_2O and 50% H_2O mixture. (One Correlation Length and Power Law)

	**MOF-919-Wet**
**Porod Scale**	2.20E-06
**Porod Exponent**	3.23
**Correlation Length Scale**	266.88
**Correlation Length [Å]**	252.58
**Exponent m**	6.60
**Bkg [1/cm]**	0.19

Supplementary Table 3. The fitting parameters of GFP@MOF-919 (**C1-C4**) using two correlation length model in the contrast matching point of 50%/ 50% D_2O / H_2O mixture.

	**C1**	**C2**	**C3**	**C4**
**Coefficient, A**	9.16E-05	7.64E-05	6.35E-05	3.93E-05
**(-) Power**	2.81	2.83	2.84	2.89
**Incoherent Bgd (cm^{-1})**	0	0	0	0
**#1 Correlation Length scale**	729.89	17.40	126.10	8.04
**#1 Correlation length [Å]**	226	226	226	226
**#1 exponent m**	6.81	6.81	6.81	6.81
**#2 Correlation Length scale**	0.92	0.26	0.08	0.12
**#2 Correlation length [Å]**	22.82	21.93	19.02	17.02
**#2 exponent m**	5.36	4.84	4.11	6.14
**Bkg [1/cm]**	0.16	0.17	0.16	0.16

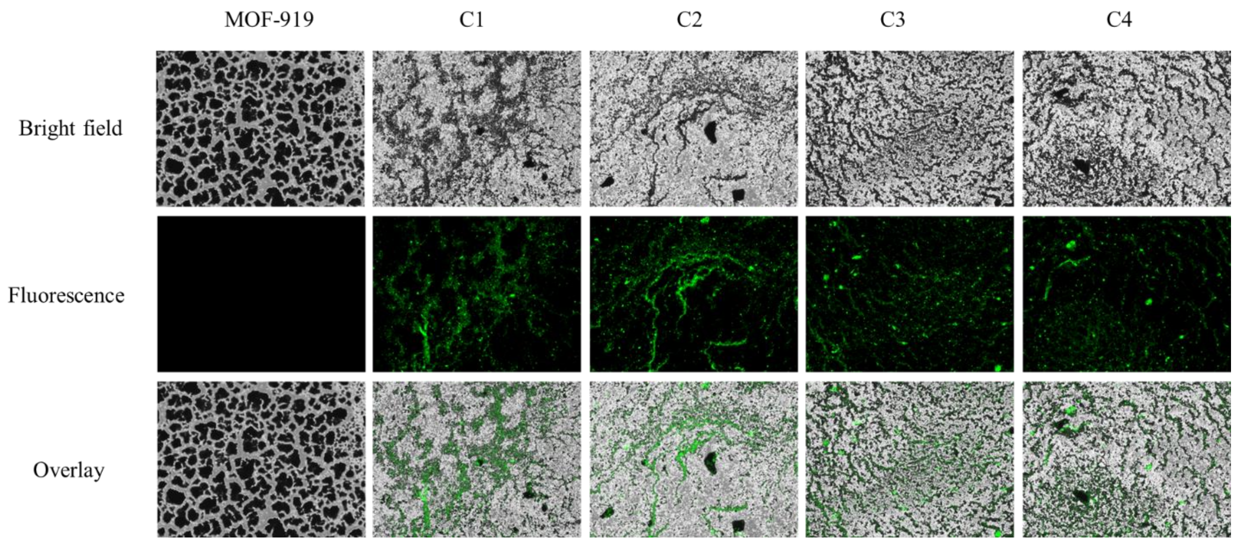

Supplementary Figure 7. Fluorescent microscopy images of MOF-919 before and after the inclusion of d-GFP (**C1-C4**) under bright field, fluorescence, and overlay.

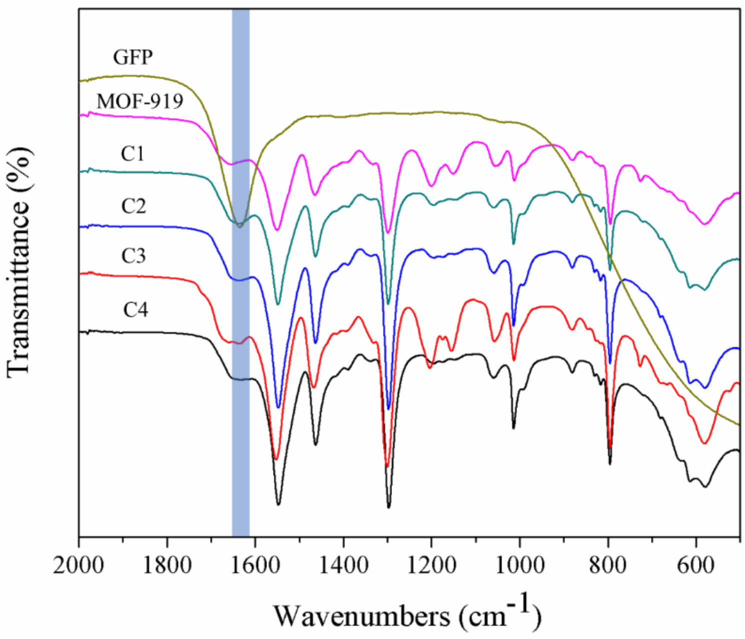

Supplementary Figure 8. FT-IR spectra of free d-GFP and MOF-919 before and after the inclusion of d-GFP (**C1-C4**).

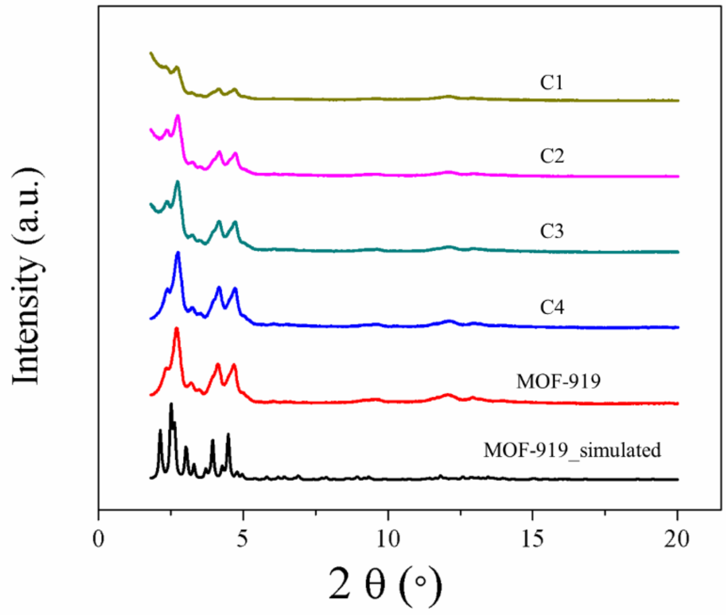

Supplementary Figure 9. PXRD of MOF-919 before and after inclusion of d-GFP (**C1-C4**) in 20 mM Tris-buffer at pH 7.5.

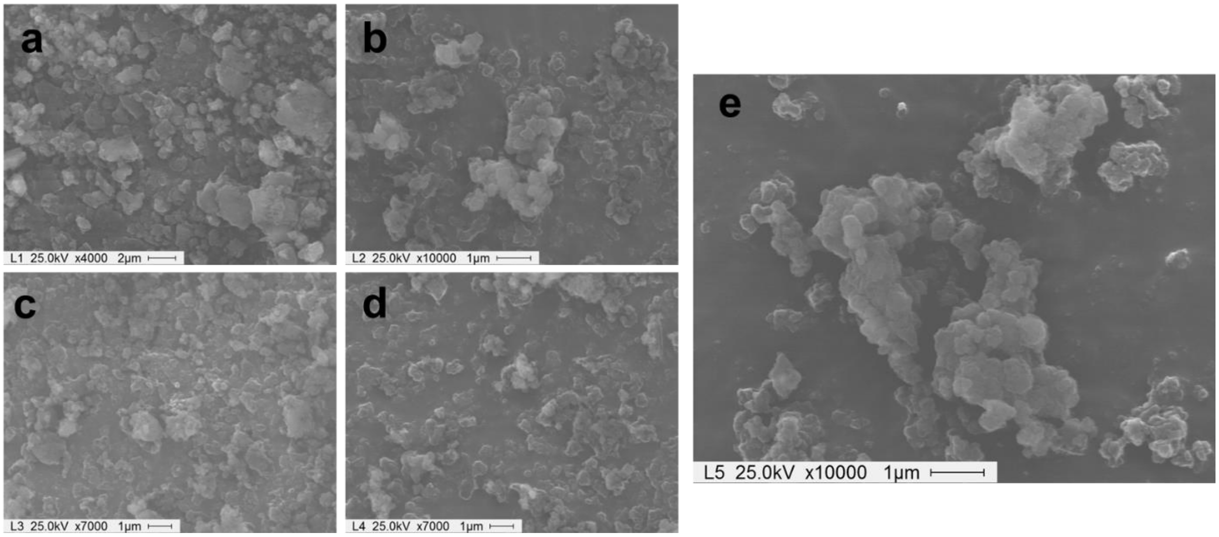

Supplementary Figure 10. SEM of (a-d) MOF-919 after inclusion of d-GFP (**C1-C4**) in 20 mM Tris-buffer, pH 7.5 and (e) unloaded MOF-919.

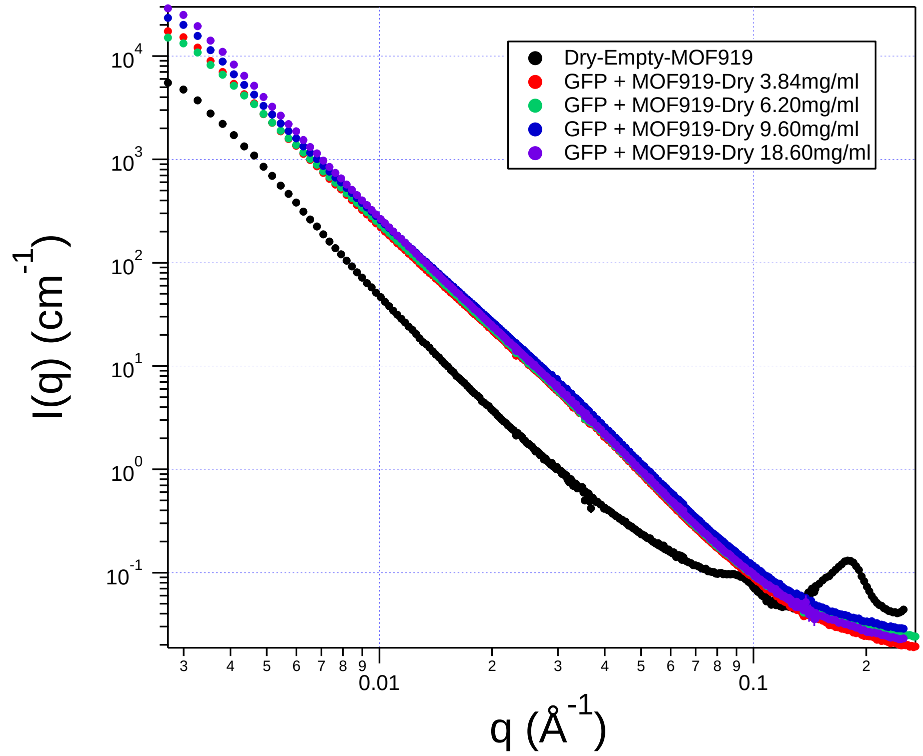

Supplementary Figure 11. Scattering profiles of dry MOF-919 and MOF-919 loaded with d-GFP as a function of protein concentration. A hump develops at ~ 0.04 A^{-1} with the entering of d-GFP.

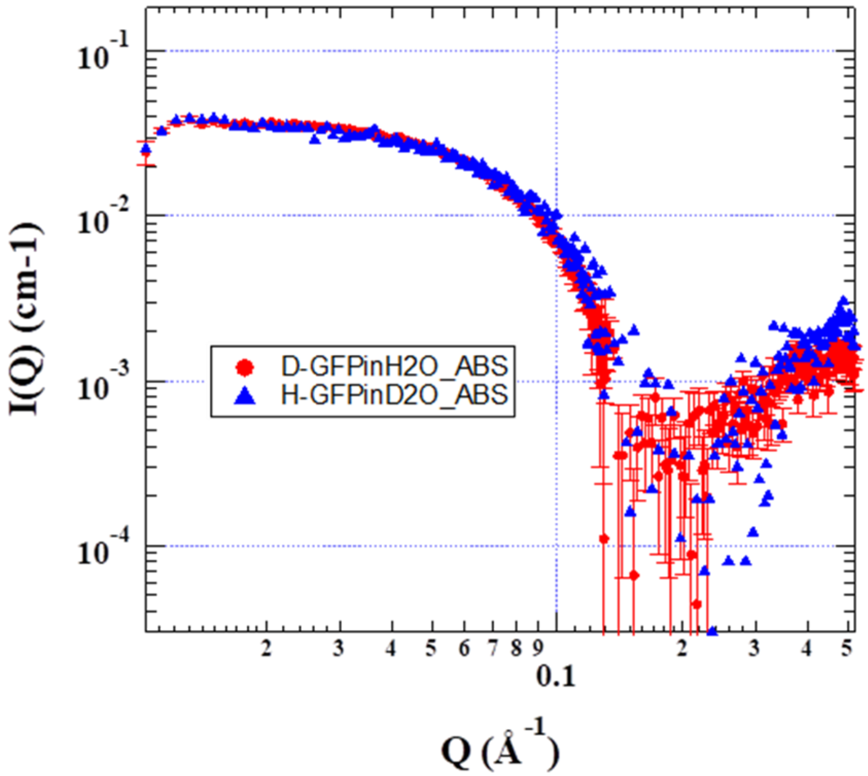

Supplementary Figure 12. Scattering profiles of free d-GFP in 50%/50% D_2O/H_2O, and free h-GFP in 95%/5% D_2O/H_2O.

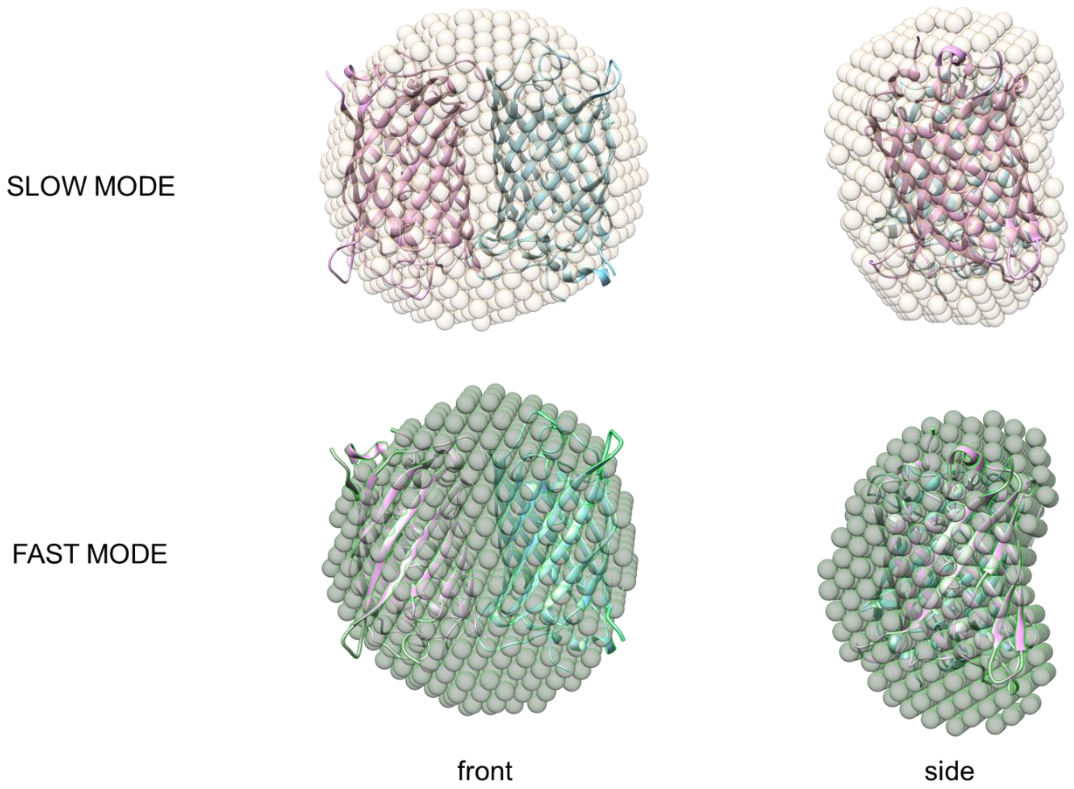

Supplementary Figure 13. DAMMIF reconstruction (as shown in light gold and dark green beads) of d-GFP in H_2O (50%/50% D_2O/H_2O) with fast and slow mode. The PDB structure is overlaid for validation.

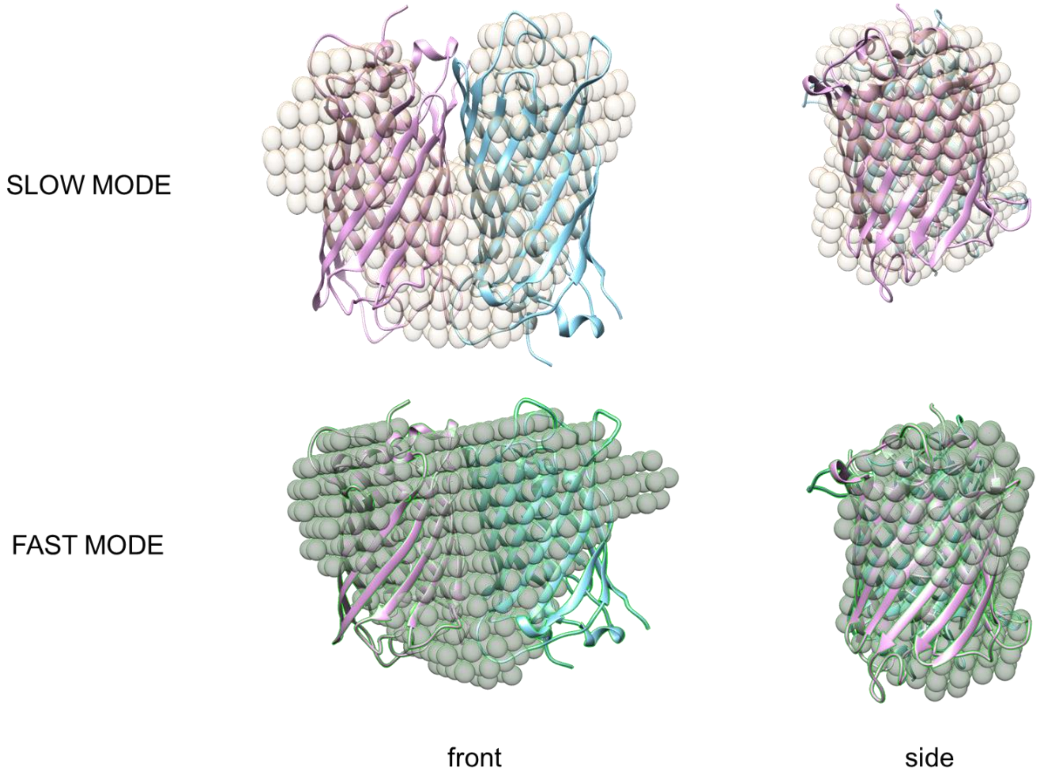

Supplementary Figure 14. DAMMIF reconstruction of h-GFP (as shown in light gold and dark green beads) in D_2O (95%/5% D_2O/H_2O) with fast and slow mode. The PDB structure is overlaid for validation.

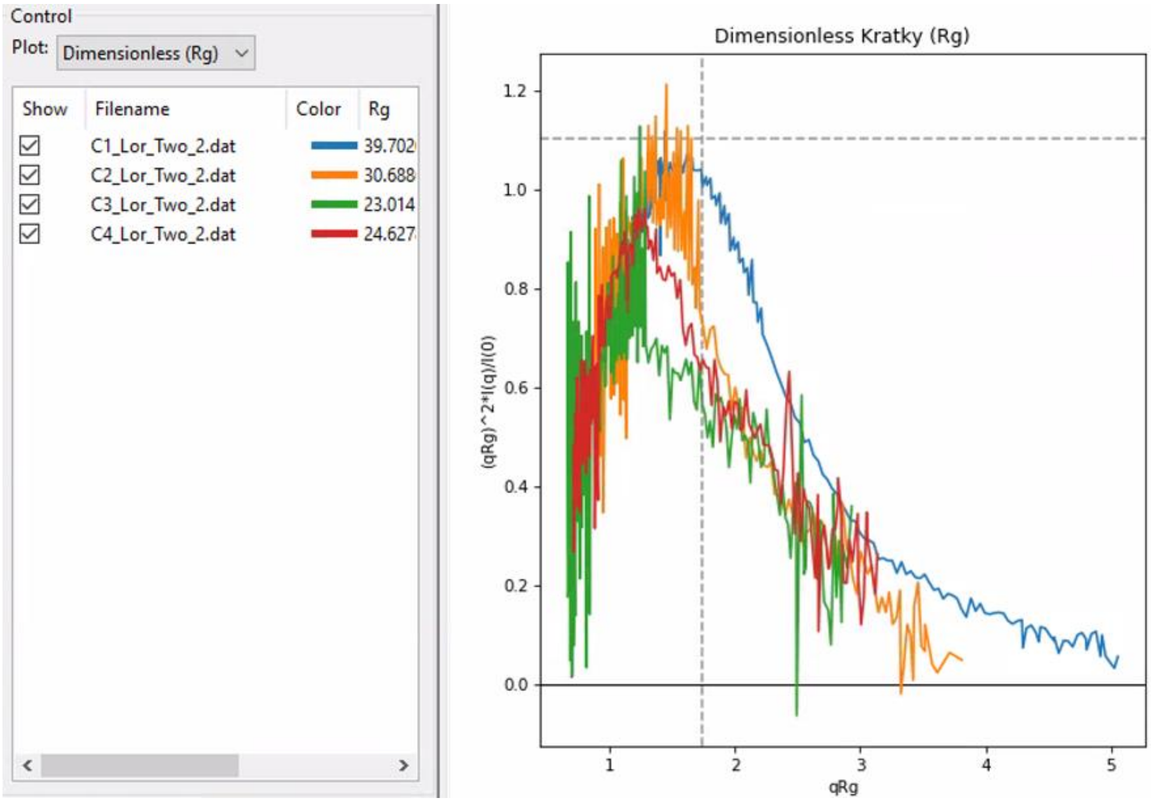

Supplementary Figure 15. Kratky plot for d-GFP loaded in the MOF-919 (**C1-C4**).

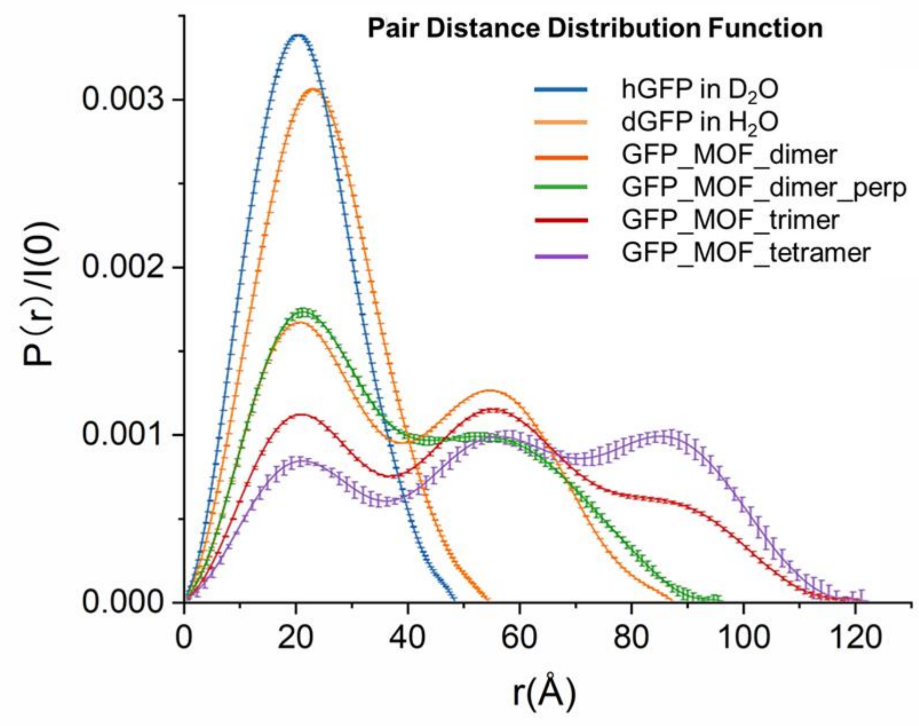

Supplementary Figure 16. P(r) curves for d-GFP in the MOF-919.

Supplementary Table 4. Parameters of calculated for multimer simulations curves created with CRYSON using d-GFP and 50% H_2O:50% D_2O to mimic experimental conditions.

	**Peak a (Å)**	**Peak b (Å)**	**Peak c (Å)**	**Rg (Å)**	**Dmax (Å)**
**Monomer**	20.78	-	-	16.82	49
**Dimer (Side-by-side)**	21.11	55.08	-	30.73	88
**Dimer (Perpendicular)**	21.69	53.72	-	31.06	97
**Trimer**	21.49	56.05	87.11	40.75	122
**Tetramer**	21.49	56.83	86.14	46.04	123
**h-GFP in D_2O**	23.62	-	-	18.90	55

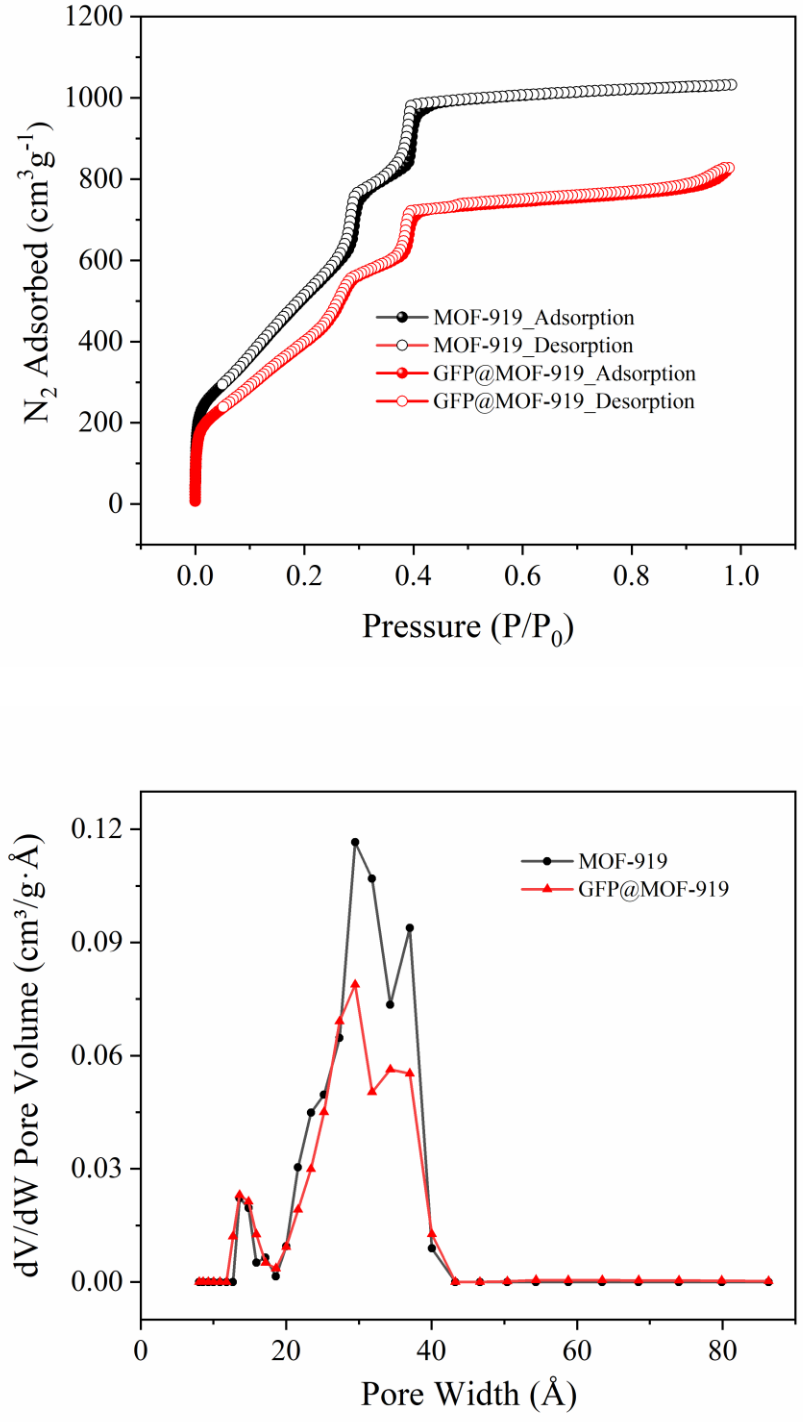

Supplementary Figure 17. N_2 adsorption isotherms at 77 K of MOF-919 before and after inclusion of GFP, and pore dimensions based on the N_2 adsorption isotherms.

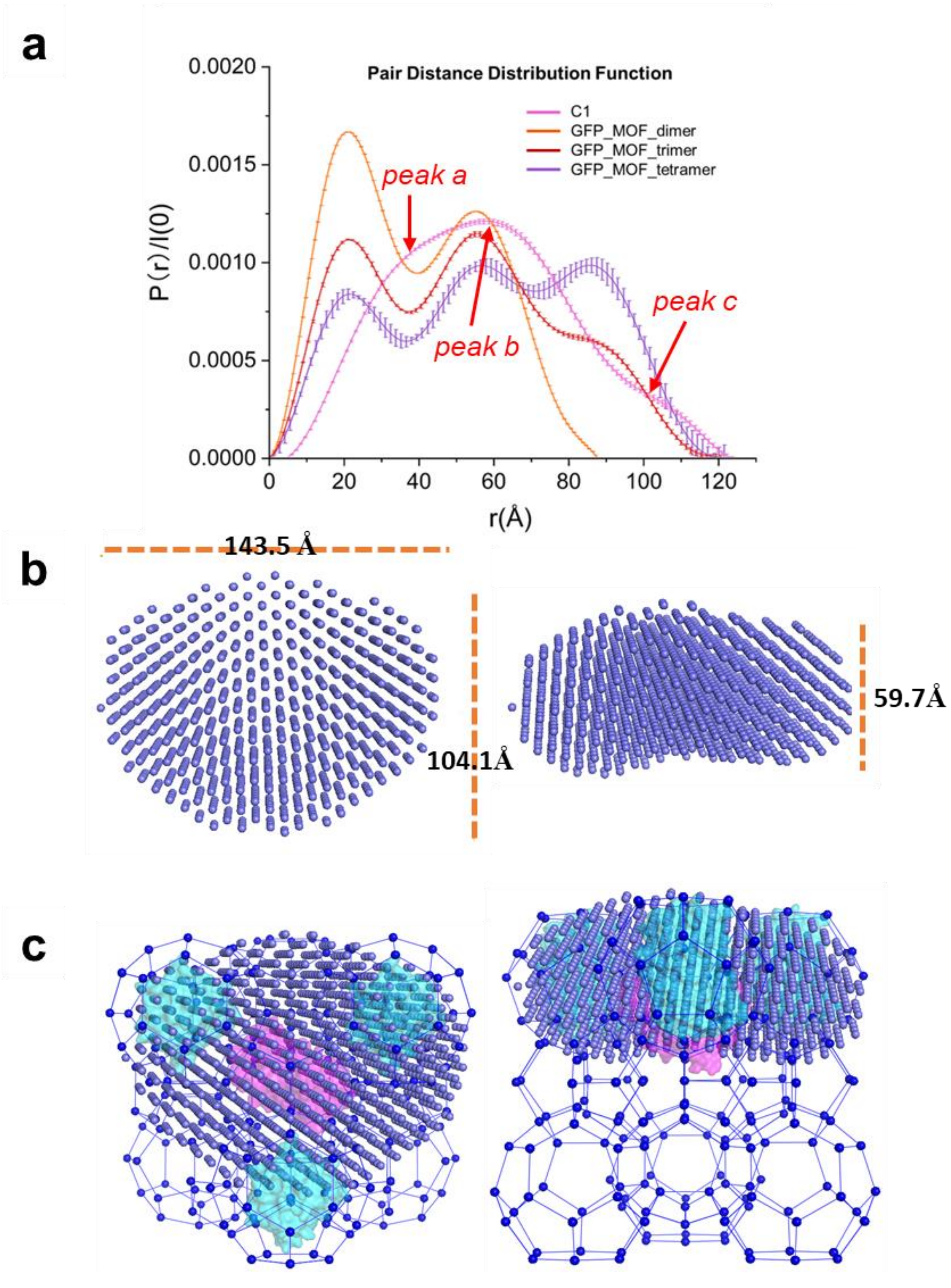

Supplementary Figure 18. (a) Comparison of calculated and experimental P(r) curves of d-GFP in MOF-919. Peaks or hump of **C1** are indicated by red arrow. (b) DAMMIF model and (c) 3D reconstruction of **C1** in structure of MOF-919.

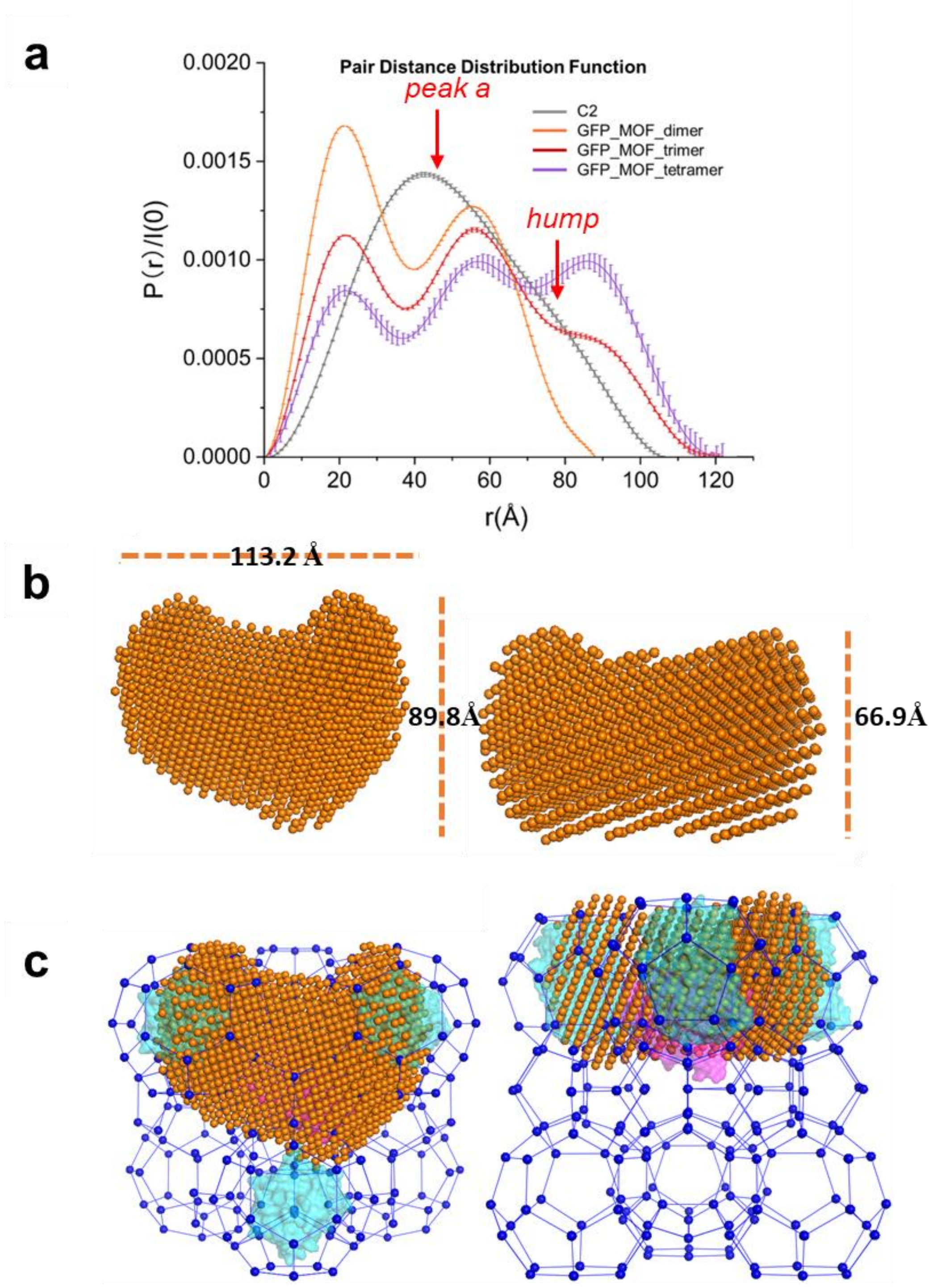

Supplementary Figure 19. (a) Comparison of calculated and experimental P(r) curves of d-GFP in MOF-919. Peak or hump of **C2** is indicated by red arrow. (b) DAMMIF model and (c) 3D reconstruction of **C2** in structure of MOF-919.

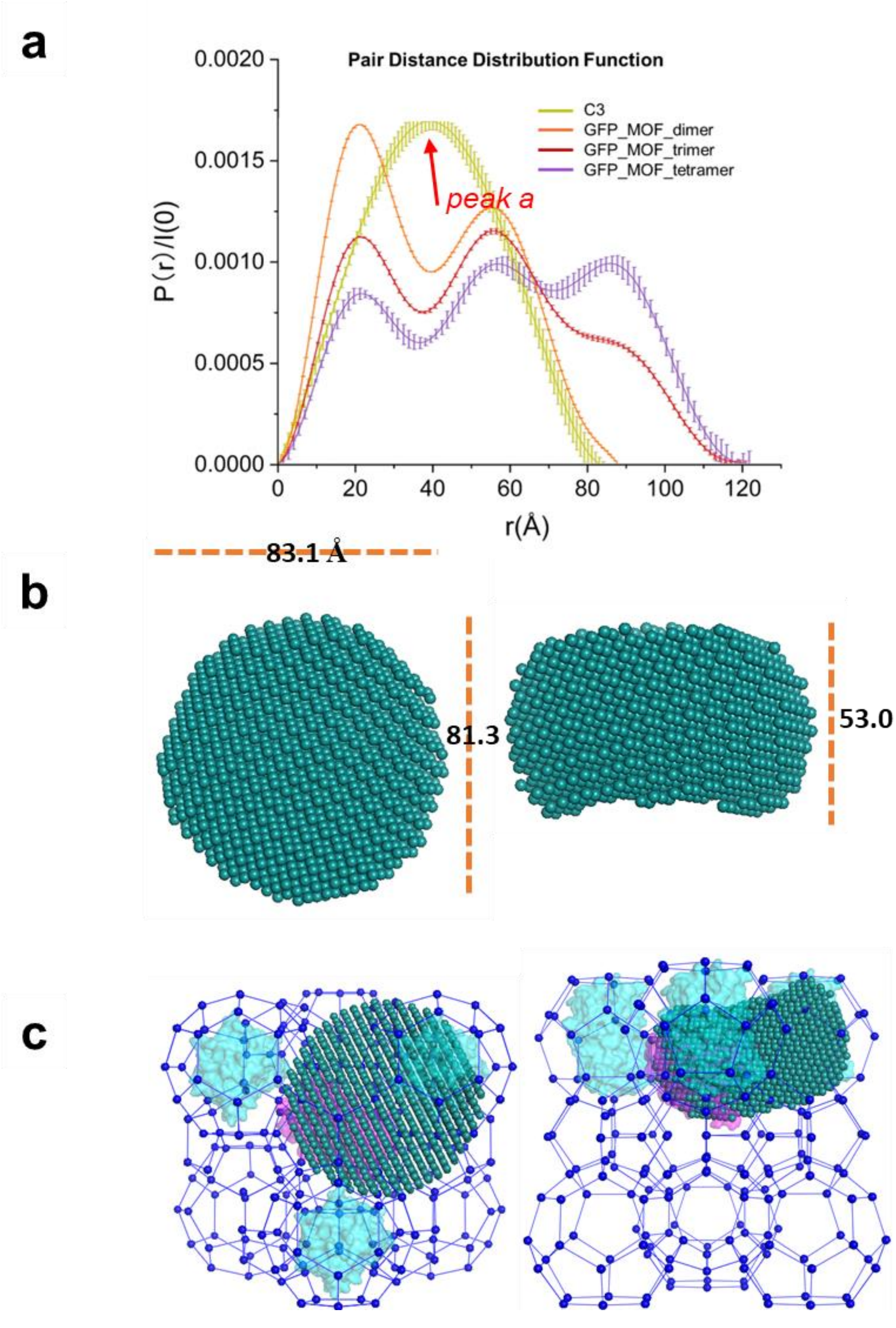

Supplementary Figure 20. (a) Comparison of calculated and experimental P(r) curves of d-GFP in MOF-919. Peak of **C3** is indicated by red arrow. (b) DAMMIF model and (c) 3D reconstruction of **C3** in structure of MOF-919.

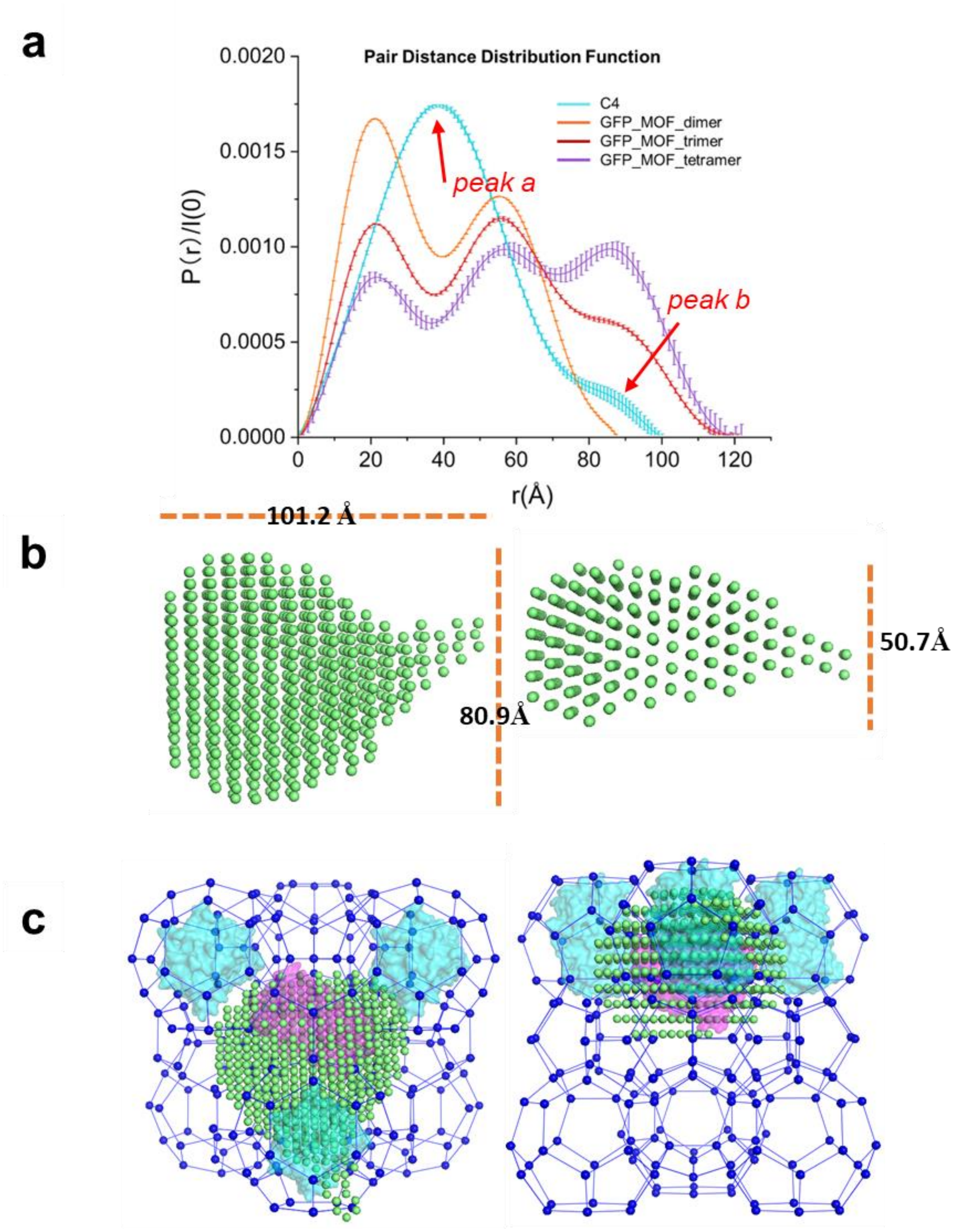

Supplementary Figure 21. (a) Comparison of calculated and experimental P(r) curves of d-GFP in MOF-919. Peaks of **C4** are indicated by red arrow. (b) DAMMIF model and (c) 3D reconstruction of **C4** in structure of MOF-919.

Supplementary Table 5. Porod Volume data for the simulated and experimental data (calculated using RAW's Molecular Weight feature)

No.	Name	Volume (Å^3)	Ratio to 1	Ratio to 2	Ratio to 3	Ratio to 4	Ratio to 5	Ratio to 6	Ratio to 7	Ratio to 8	Ratio to 9	Ratio to 10	Ratio to 11	Ratio to 12
1	dGFP (H_2O)	30200	1.00	0.83	0.57	0.57	0.37	0.27	0.10	0.16	0.32	0.30	0.49	0.82
2	hGFP (D_2O)	36500	1.21	1.00	0.69	0.69	0.45	0.32	0.12	0.20	0.39	0.37	0.60	0.99
3	Dimer	52800	1.75	1.45	1.00	0.99	0.65	0.47	0.17	0.29	0.56	0.53	0.86	1.43
4	Dimer Perp	53100	1.76	1.45	1.01	1.00	0.66	0.47	0.17	0.29	0.56	0.53	0.87	1.44
5	Trimer	80700	2.67	2.21	1.53	1.52	1.00	0.71	0.26	0.44	0.86	0.81	1.32	2.19
6	Tetramer	113000	3.74	3.10	2.14	2.13	1.40	1.00	0.36	0.61	1.20	1.13	1.85	3.06
7	C1	310000	10.26	8.49	5.87	5.84	3.84	2.74	1.00	1.68	3.29	3.11	5.07	8.40
8	C2	185000	6.13	5.07	3.50	3.48	2.29	1.64	0.60	1.00	1.97	1.86	3.03	5.01
9	C3	94100	3.12	2.58	1.78	1.77	1.17	0.83	0.30	0.51	1.00	0.94	1.54	2.55
10	C4	99700	3.30	2.73	1.89	1.88	1.24	0.88	0.32	0.54	1.06	1.00	1.63	2.70
11	dGFP in H_2O	61100	2.02	1.67	1.16	1.15	0.76	0.54	0.20	0.33	0.65	0.61	1.00	1.66
12	hGFP in D_2O	36900	1.22	1.01	0.70	0.69	0.46	0.33	0.12	0.20	0.39	0.37	0.60	1.00

The Porod volumes for the simulated data scale approximately with how many particles comprise the shape (~30,000 Å^3 per GFP molecule). No. 1-6 are simulated based on crystal structure of dGFP and hGFP, and No. 7-12 are based on experimental results.
